# Comparing a head‐mounted virtual reality perimeter and the Humphrey Field Analyzer for visual field testing in healthy and glaucoma patients

**DOI:** 10.1111/opo.13229

**Published:** 2023-10-06

**Authors:** Jack Phu, Henrietta Wang, Michael Kalloniatis

**Affiliations:** ^1^ School of Optometry and Vision Science University of New South Wales Sydney Kensington New South Wales Australia; ^2^ Centre for Eye Health University of New South Wales Sydney Kensington New South Wales Australia; ^3^ Faculty of Medicine and Health University of Sydney Camperdown New South Wales Australia; ^4^ Concord Clinical School Concord Repatriation General Hospital Concord New South Wales Australia; ^5^ School of Medicine (Optometry) Deakin University Waurn Ponds Victoria Australia

**Keywords:** mesopic, portable perimetry, SITA, SITA‐Faster, standard automated perimetry, thresholding, VR

## Abstract

**Purpose:**

To compare clinical visual field outputs in glaucoma and healthy patients returned by the Humphrey Field Analyzer (HFA) and virtual reality (Virtual Field, VF) perimetry.

**Methods:**

One eye of 54 glaucoma patients and 41 healthy subjects was prospectively tested (three times each in random order) using the HFA and VF perimeters (24‐2 test grids). We extracted and compared global indices (mean deviation [MD] and pattern standard deviation [PSD]), pointwise sensitivity (and calculated ‘equivalent’ sensitivity after accounting for differences in background luminance) and pointwise defects. Bland–Altman (mean difference [*M*_diff_] and 95% limits of agreement [LoA]) and intraclass correlation analyses were performed.

**Results:**

The VF test was shorter (by 76 s) and had lower fixation losses (by 0.08) and false‐positive rate (by 0.01) compared to the HFA (all *p* < 0.0001). Intraclass correlations were 0.86, 0.82 and 0.47 for MD, PSD and pointwise sensitivity between devices, respectively. Test–retest variability was higher for VF (*M*_diff_ 0.3 dB, LoA −7.6 to 8.2 dB) compared to the HFA (*M*_diff_ −0.3 dB, LoA −6.4 to 5.9 dB), indicating greater test–retest variability. When using each device's underlying normative database, the HFA detected, on average, 7 more defects (at the *p* < 0.05 level) out of the 52 test locations compared to this iteration of VF in the glaucoma cohort.

**Conclusions:**

Virtual Field returns global results that are correlated with the HFA, but pointwise sensitivities were more variable. Differences in test–retest variability and defect detection by its current normative database raise questions about the widespread adoption of VF in lieu of the HFA.

**Key points**

- Virtual reality‐based perimetry using the Virtual Field device returned global indices that correlated with the Humphrey Field Analyzer.
- Pointwise variability and test–retest variability were found to be greater with the Virtual Field compared to the Humphrey Field Analyzer.
- There were fewer defects detected by the Virtual Field in comparison with the Humphrey Field Analyzer when using their respective commercially available normative databases.

## INTRODUCTION

In‐office standard automated perimetry remains a mainstay in glaucoma management for quantifying visual function and staging and prognosticating the disease.^1^, ^2^, ^3^ However, in‐office devices have several known limitations, including cost, size (office space) and patient discomfort during the test.^4^ In principle, standard automated perimetry is a psychophysically simple procedure, requiring only one button press in response to a visual stimulus, and it can be ported over to an alternative testing platform. Consumer‐friendly technologies, such as tablets and virtual reality (VR) headsets that are portable, affordable and comfortable, have been proposed to address the limitations of in‐office equipment.^4^, ^5^ This problem was specifically highlighted during the COVID‐19 pandemic in the context of limited resources, space and staff.^6^, ^7^

Several advantages of VR headsets compared to other testing methods have been proposed.^8^ They have immersive screens that encompass the visual field and can potentially reduce the effects of external sources of straylight and visual distractors (though internal reflections are likely to remain). Headsets can maintain a fixed working distance and incorporate refractive correction with less patient input required, in comparison to tablet based technology. Internal gyroscopic mechanisms can compensate for the user's head position so patients can perform the test in their preferred sitting position.

However, there are also some potential disadvantages to screen‐based perimetry. A critical review by Ma et al.^8^ highlighted the lack of standardisation among hardware and software parameters that introduces substantial heterogeneity among different products. Specifically, although internal gyroscopic mechanisms can compensate for head movements, these may in turn introduce stimulus jitter. Furthermore, commonly used VR headsets incorporate the use of Fresnel lenses that require correction of introduced distortion, which was a topic of recent investigation.^9^

Majorly, unlike projection perimetry, such as that performed using the Humphrey Field Analyzer (HFA), there are limits to the dynamic range of the instrument due to the lower maximum luminance output of the screen.^3^, ^10^ To expand the dynamic range, the background luminance of screen‐based devices often needs to be reduced, or to reduce the stimulus relative to the background (i.e., stimulus decrements, such as that demonstrated by Anderson and Vingrys^11^). Specifically, reductions in background luminance may mean that some perimeters assess the visual pathway under different retinal adaptation conditions (such as mesopic or scotopic, which would therefore also progressively involve the rod pathway with decreasing background luminance). Since perimetric data are conventionally reported in attenuation (decibels, dB), the output value is a quantity derived from the stimulus luminance relative to its background and maximal level. Accordingly, to obtain sufficient steps, each incremental change in stimulus luminance needs to be adjusted by the background and maximal luminance. As such, the sensitivity outputs are not necessarily directly comparable between devices.

Despite these limitations, VR testing platforms may play a role in perimetric testing in clinical practice, such as improving accessibility in low‐resource settings. With increasing demands on office time and space, and with increasing interest in home monitoring strategies, there is growing interest in evaluating the suitability of these technologies for clinical deployment.

Several studies have examined VR‐based perimeters. For example, Shetty et al.^12^ and Mees et al.^13^ showed that VR platforms may be useful when screening for glaucomatous visual field defects using the criteria of number of defective points identified. Other studies using different thresholding paradigms, such as that by Narang et al.,^14^ Razeghinejad et al.^15^ and Tsapakis et al.^16^ against the HFA and Stapelfeldt et al.^17^ against the Octopus, showed results that correlated well with standard automated perimetry. Hu et al.^18^ showed that home visual field tests using VR is acceptable and feasible among glaucoma suspects and patients with glaucoma.

One device that is approved by the US Food and Drug Administration (FDA) as a class I medical device for performing perimetry is the Virtual Field (VF; Virtual Field Inc., virtualfield.io/). To date, there has been only one published study reporting on its deployment in clinical practice,^18^ but to our knowledge, there has been no comprehensive published report on comparisons of important perimetric parameters such as mean deviation and pointwise sensitivity. In the present study, we evaluated an iteration of the VF, in its commercial, clinician‐facing state, for examining the visual fields of healthy and open angle glaucoma subjects in comparison to the HFA. The main variables of interest were output sensitivities, pattern deviation probability scores and global indices.

## METHODS

This was a prospective, cross‐sectional study. Ethics approval was provided by the Human Research Ethics Committee of the University of New South Wales. The study adhered to the tenets of the Declaration of Helsinki. Subjects provided written‐informed consent prior to inclusion in the study.

### Subject cohort

Ninety‐five subjects in the present study were recruited from the Centre for Eye Health (University of New South Wales) general and glaucoma clinics and among staff and their family members.

The diagnosis of open angle glaucoma was made as per current clinical guidelines^19^ and per the criteria detailed in our previous studies.^20^, ^21^ In brief, structural evidence of glaucoma included enlarged or asymmetric cup‐to‐disc ratio, diffuse or focal rim thinning and adjacent retinal nerve fibre layer defects that were not explained by other retinal or neurological pathologies found on clinical examination supplemented with ocular imaging (colour fundus photography and optic nerve head and macular optical coherence tomography). Visual field defects were not required for a diagnosis of open angle glaucoma, that is, subjects with pre‐perimetric glaucoma were also included if otherwise eligible. Subjects who had other co‐morbid diseases of the visual pathway, such as age‐related macular degeneration or a neurological insult (confirmed using neuroimaging), were excluded from the study. The glaucoma cohort included patients across a variety of intraocular pressure strata, and all had open angles on gonioscopy.

For healthy subjects, we prospectively recruited from the general clinic and among staff and their family members to obtain an age‐similar cohort for comparison with the glaucoma group. All subjects underwent a comprehensive eye examination, including visual acuities, intraocular pressures (measured using applanation tonometry), anterior chamber angle evaluation, corneal thickness, anterior and posterior segment physical examination on the slit lamp biomicroscope and supplemented using ocular imaging as described above. Subjects in whom there was no evidence of diseases in the visual pathway (retinal, optic nerve or neurological) and with intraocular pressures <21 mmHg were eligible for inclusion.

Exclusion criteria for both glaucoma and healthy cohorts were: age <18 years, best corrected visual acuities worse than 0.18 logMAR (6/9), spherical equivalent refractive error outside the range of +6.00 to −8.00 D, history of ocular trauma or surgery (aside from uncomplicated cataract surgery, selective laser trabeculoplasty or laser peripheral iridotomy performed at least 3 months prior) and a physical or cognitive impediment that precluded perimetric testing. Perimetric experience was not required; however, as a product of recruiting largely from the clinical service, nearly all patients had previously performed standard automated perimetry.

### Visual field testing

Both glaucoma and healthy subjects underwent visual field testing using the HFA (as the standard automated perimetry reference test) and the VF on the same day. Each subject underwent testing at least three times using each device, and testing was performed in random order (by test, i.e., no bias in terms of which was done first) for all subjects with rest breaks in between each test as required. All subjects underwent a practice test run for both tests if they had no prior perimetric experience to ensure task understanding. The practice test was not included in the analysis. All testing was conducted by one of the study investigators or a trained research assistant.

Testing on the HFA (HFA3; Carl Zeiss Meditec, zeiss.com/meditec/en/products/perimetry/humphrey‐field‐analyzer‐3.html) was performed on the 24‐2 test grid using the Swedish Interactive Thresholding Algorithm (SITA) standard. A Goldmann size III stimulus (0.43° diameter) was used with the default background luminance setting of 10 cd.m^−2^. The maximum output luminance of the HFA is 3183 cd.m^−2^. The subject's refractive error was entered into the instrument and an appropriate trial lens was placed into the bracket to ensure a clear retinal image. Fixation was monitored using the live infrared camera by the operator. Despite recent debate regarding the application of automated reliability indices for assessing test reliability,^22^, ^23^, ^24^, ^25^ the automatic outputs by the devices remain used clinically, and thus, we extracted these for the present study, including fixation losses (using the Heijl–Krakau blind spot monitoring method^26^), false‐positive rate, false‐negative rate and the gaze tracker.

The VF is a VR‐based perimetry software and, in the present study, was performed using an Oculus Go device (Meta Reality Labs, oculus.com/experiences/go/). The software is FDA approved for use as an automated perimeter. A Goldmann size III stimulus was used for this study, and the presentation time was the same as the HFA (200 ms). Due to the physical range limitations of the device, the background luminance was 0.218 cd.m^−2^, with a maximum stimulus luminance output of 87 cd.m^−2^. The background luminance represents a fundamental difference between devices. The HFA uses a background luminance of 10 cd.m^−2^, which puts it into the low photopic range. Despite the lower background luminance, the extent to which rods contribute to detection over the cones is questionable, as described by experiments by Lauritzen et al.^27^ More recently, work by Simunovic et al.^28^ demonstrated that under mesopic light levels, detection of Goldmann size III targets is also predominantly mediated by the cones. As we were assessing a device that is commercially available by a private manufacturer, we had no control over the device's background luminance parameters. This is discussed further in the Discussion section.

Reliability metrics are similarly output by the device (fixation losses, false‐positive rate and false‐negative rate), except for the gaze tracker. The methods for determining these reliability metrics are similar to that of the HFA. The VF has several thresholding algorithms available for testing. In the present study, we used the Fast Full Threshold algorithm.

For each subject with glaucoma, the eye with glaucoma was chosen for the study, and if both eyes had glaucoma, one was randomly selected. For healthy subjects, a random eye was selected for testing.

### Visual field outputs: Global indices, reliability indices and pointwise sensitivity

Both the HFA and the VF devices provide printouts useful for clinical decision making, including maps such as the greyscale, sensitivity plot, total deviation, pattern deviation and their probability scores. We specifically extracted the pointwise sensitivity values and the pointwise pattern deviation probability scores for analysis. Global indices (mean deviation and pattern standard deviation) and reliability indices (fixation losses, false‐positive rate and false‐negative rate) and test duration were also extracted for analysis.

Global sensitivity metrics were compared using correlation and Bland–Altman analyses. We also calculated intraclass correlations for the variables of interest, as per the work of Prea et al.^29^ Although there are differences in how mean deviation and pattern standard deviation are calculated for each device (as they use reference normative data and additional proprietary weightings at each test location), these are clinician‐facing indices, and their direct comparisons remain interesting.

Although the isolated clinical use of reliability indices such as fixation loss, false‐positive and false‐negative rate is debated in the literature,^22^, ^23^, ^24^, ^25^ these remain the sole clinician‐facing indices in perimetry printouts. In the present study, we compared the numerical values of the output reliability indices as they use similar methodologies, but did not use specific cut‐off criteria to exclude tests as unreliable.

### Visual field outputs: Pointwise sensitivity

We extracted pointwise sensitivity from the sensitivity maps using both devices. These were the raw values prior to modification for the pattern deviation plots that are reported on the clinician‐facing printout. The dB value was averaged for each subject at each test location across the three tests, and direct comparisons were performed between devices using the same correlation and Bland–Altman methods as described for the global indices. Test–retest variability was assessed by taking the second and third visual field test results for each patient and performing both Bland–Altman and correlation analyses. In addition to Bland–Altman comparisons for test–retest variability, we also calculated the coefficient of reliability (CR) using the following equation as per the recommendations of Bland and Altman.^30^

$$\mathrm{CR}=1.96\times \sqrt{\frac{\sum {\left(d_{2}-d_{1}\right)}^{2}}{n}}$$

where *d* represents the variable (such as mean deviation) from the first and second tests of interest and *n* indicates the sample size.

Additionally, we also performed pointwise sensitivity analyses after excluding test locations that were below the HFA measurement floor, as described in earlier studies^31^, ^32^ and our previous work.^20^, ^21^, ^24^ This is because the test–retest variability is much larger below the floor, which introduces heteroscedasticity, and in practical terms, such points confound visual field interpretation and are not clinically useful.

In the present study, we report the decibel values as currently indicated in clinician‐facing outputs, as this is what is most commonly used in day‐to‐day practice. In Appendix S1, we provide additional analysis where we account for the differences in maximum output and background luminance of the respective instruments.

### Visual field outputs: Pointwise probability scores

A *p*‐value < 0.05 on the pattern deviation probability map was deemed as a significant pointwise defect for the purposes of the study. We elected to use pattern deviation, rather than total deviation, as this map is conventionally used to identify significant visual field defects in the context of glaucoma after accounting for the patient's individual Hill of Vision.^33^ Although the utility of the pattern deviation diminishes with very advanced visual field loss, this study focussed on early and moderate stages of glaucoma where the use of the pattern deviation map remains relevant. We did not use cluster criteria for this analysis, nor did we restrict the analysis to non‐edge points, as we later mapped out differences between test methods in a pointwise manner. We tested two conditions: first, when at least one of the patient's visual field results demonstrated a defect at *p* < 0.05 or lower on the pattern deviation map; and secondly, when at least two out of three of the patient's results demonstrated a defect (that was repeatable) at the same location. We binarised whether a defect was present or absent at each specific location. The number of visual field defects was calculated for each patient. Then, for each location, we determined whether there was agreement in defect (or no defect) detection at that location across the cohort. A McNemar test^34^ was performed for each test location, since there could be locations of defect or normality that were mutually identified by each technique. Discordance was then identified as either favouring the HFA or the VF, that is, one technique identifying more defects than the other.

However, given differences between the devices' underlying normative databases, we also developed a normative database using our healthy subjects (Figures S2 and S3). Since this analysis was derived using a smaller cohort, we report these results in Figure S4.

Statistical analyses in this paper were performed using Microsoft Excel (Microsoft Corporation, microsoft.com/en‐au/microsoft‐365/excel) and GraphPad Prism version 10 (GraphPad Software, graphpad.com/features).

## RESULTS

We assessed 41 healthy subjects (24 males, 17 females; mean age 64.8 years, SD 10.4 years, range 39.0–80.0 years) and 54 subjects with glaucoma (36 males, 18 females; mean age 63.7 years, SD 9.5 years, range 35.0–78.4 years) in the present study. There was no difference in age (*p* = 0.60) or gender distribution (*p* = 0.52) between the groups.

### Global sensitivity indices

**FIGURE 1:**
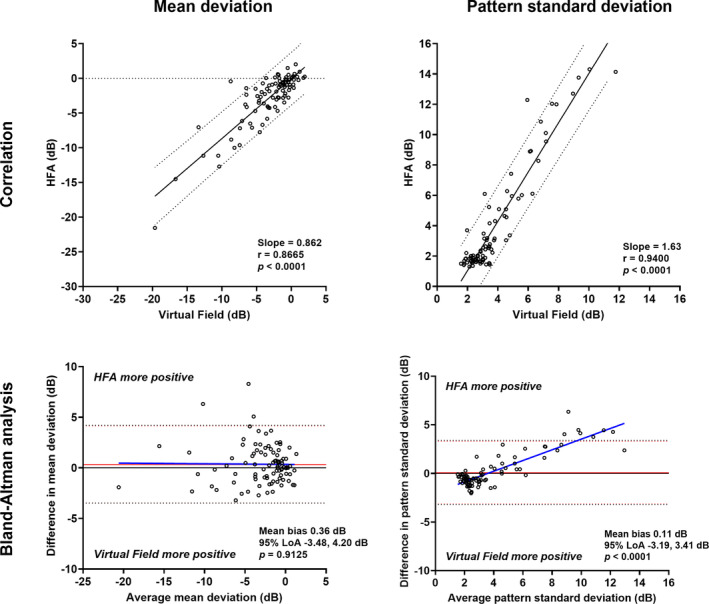
Top row: correlations between Humphrey Field Analyzer (HFA) and Virtual Field mean deviation (left) and pattern standard deviation (right) (*N* = 95). Slope, correlation coefficient and *p*‐values are shown in the inset, and the 95% prediction intervals are shown by the dashed lines. Bottom row: Bland–Altman analysis for mean deviation (left) and pattern standard deviation (right) comparing HFA and Virtual Field. The red solid line indicates the mean bias, the red dashed lines indicate the 95% limits of agreement, the blue solid line indicates the regression line (*p*‐value of the slope is shown in the inset), and the black solid line indicates *y* = 0. For the bottom panels, a more positive value indicates that the HFA returned a higher result, while a negative value indicates that the Virtual Field returned a higher result.

Instrument output mean deviation and pattern standard deviation measurements were strongly correlated between devices (Figure 1). The intraclass correlation coefficient was similar to the Pearson *r* for mean deviation (0.87 vs. 0.86) but was lower than the Pearson *r* for pattern standard deviation (0.94 vs. 0.82). The mean deviation result tended to be more negative when using the VF (or more positive on the HFA). For pattern standard deviation, the slope of the correlation suggested a more positive result on the HFA for a given VF result. There did not appear to be a systematic bias as a function of mean deviation level on the Bland–Altman analysis (mean bias 0.36 dB, 95% limits of agreement −3.48 to 4.20 dB; linear regression *p* = 0.91). The pattern standard deviation tended to be less positive when using the VF, and vice versa for the HFA. On Bland–Altman analysis, this difference appeared greater with a higher pattern standard deviation (linear regression through difference values *p* < 0.0001), indicating a systematic bias in which the HFA pattern standard deviation tended to be higher than the VF at higher levels of pattern standard deviation. The difference in pattern standard deviation between devices implies that the VF does not detect the heterogeneity of sensitivity values with advancing disease as much as the HFA (see Discussion).

### Test duration and reliability indices

On average, the HFA SITA‐Standard test took 76 s longer than the VF Fast Threshold test (*p* < 0.0001). There were higher fixation losses on the HFA (median 0.13, or 13%) compared to the VF (median 0.05, or 5%) (*p* = 0.0006). There were also higher false‐positive rates on the HFA (median 0.02, or 2%) compared to the VF (median 0.01, or 1%) (*p* < 0.0001), but this difference was much smaller than the fixation loss difference. There was no significant difference in false‐negative rate (*p* = 0.61). These are shown in Figure S5.

### Pointwise sensitivity: Comparisons between devices

**FIGURE 2:**
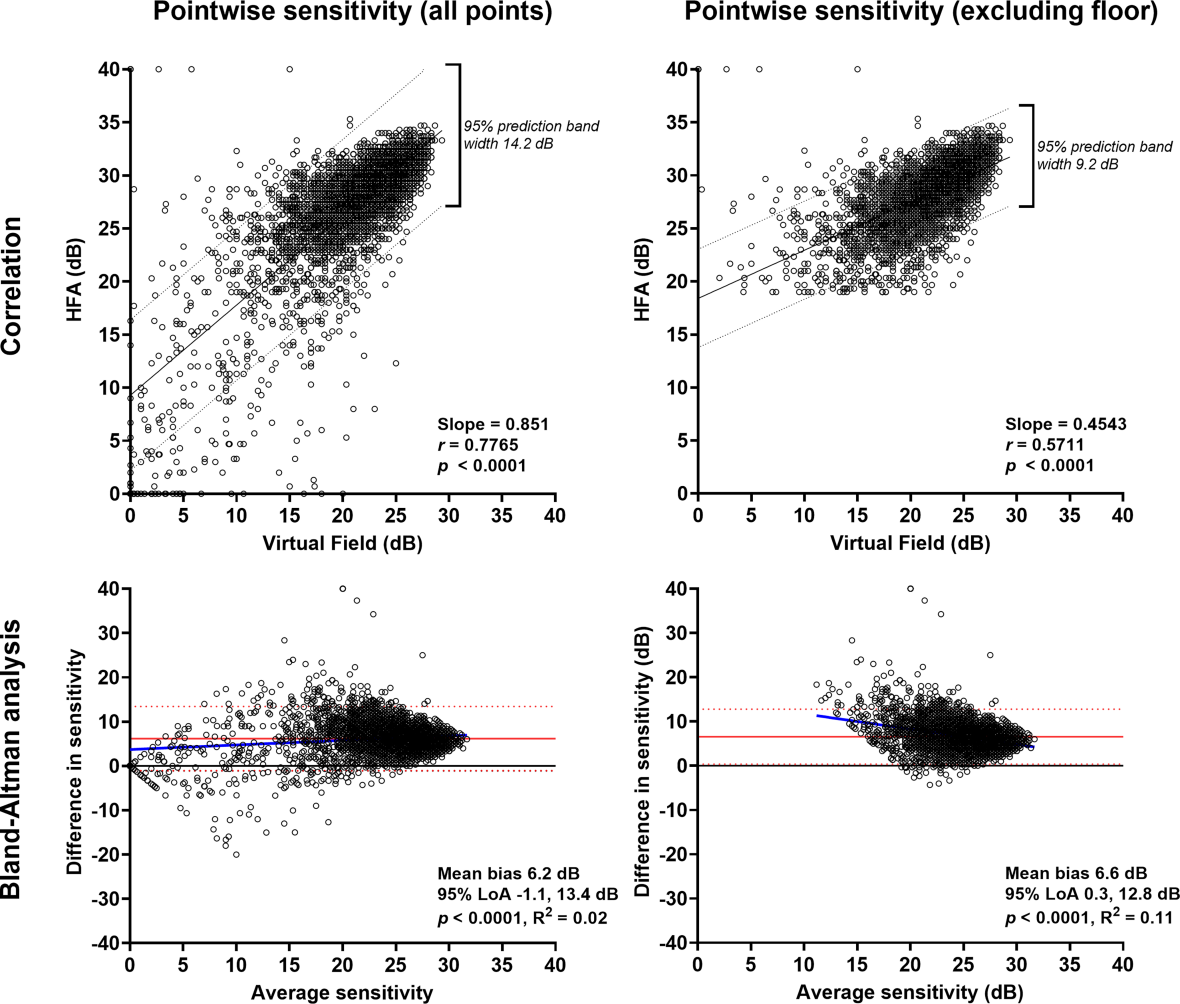
Top row: correlations between Humphrey Field Analyzer (HFA) and Virtual Field pointwise sensitivity inclusive of all points (left) (*N* = 4940) and when points reaching the HFA floor (<19 dB) were excluded (right) (*N* = 4617). Slope, correlation coefficient and *p*‐values are shown in the inset, and the 95% prediction intervals are shown by the dashed lines. Bottom row: Bland–Altman analyses for pointwise sensitivity inclusive of all points (left) and when points reaching the HFA floor (<19 dB) were excluded (right). The red solid line indicates the mean bias, the red dashed lines indicate the 95% limits of agreement, the blue solid line indicates the regression line (*p*‐value of the slope is shown in the inset) and the black solid line indicates *y* = 0. For the bottom panels, the differences were Virtual Field–HFA, such that a negative *y*‐axis value indicates a lower sensitivity value found on the Virtual Field, and vice versa.

Although the Pearson correlation in pointwise sensitivity between devices was moderate (0.78, Figure 2), the intraclass coefficient was lower at 0.47. The HFA sensitivity outputs were higher than the VF results, attributable to the differences in dynamic range. The difference in dynamic range also contributes to explaining the discordance between the Pearson and intraclass correlation values. There was substantial heteroscedasticity in the relationship with lower sensitivity values, consistent with poorer variability at those levels. Although the correlation was higher when including all sensitivity measurements compared to when those reaching the HFA floor (less than 19 dB) were excluded (*r =* 0.78 vs. *r =* 0.57; intraclass coefficient 0.47 vs. 0.34), the 95% limits of agreement were wider when all measurements were included compared to when the points below the floor were excluded (−1.1 to 13.4 dB and 0.3 to 12.8 dB, respectively) (Figure 2).

Bland–Altman analyses were consistent with these correlations, with a bias in sensitivity measurements of over 6 dB for both all points and floor‐excluded conditions. Although there was a statistically significant effect of average sensitivity on the bias (*p* < 0.0001 for both conditions), the coefficient of determination was very low, and each was inconsistent with the other, suggesting the absence of a true effect (Figure 2).

### Pointwise sensitivity: Within‐device test–retest variability

**FIGURE 3:**
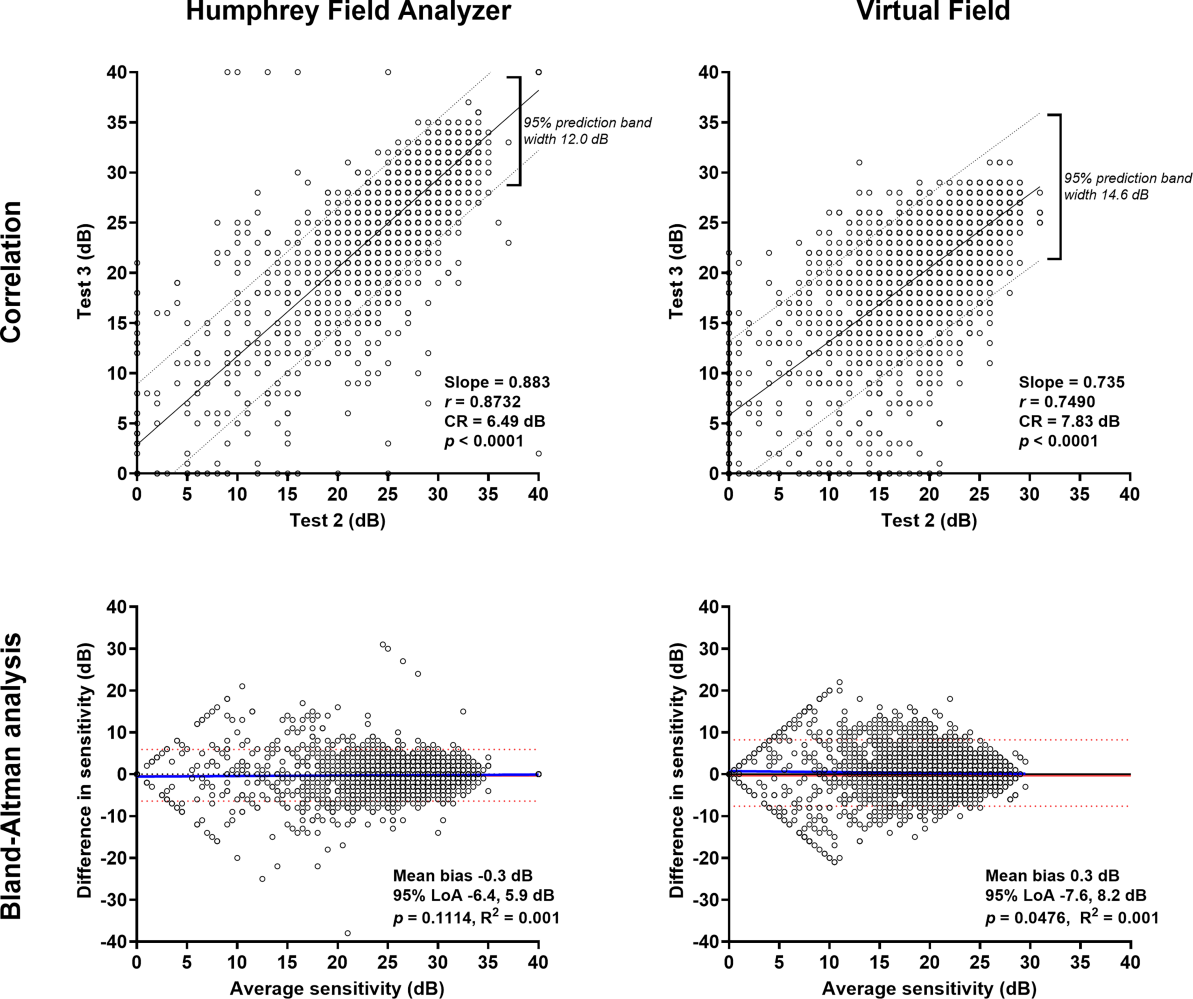
Top row: correlations between Tests 2 and 3 for the Humphrey Field Analyzer (HFA) (left) and Virtual Field (right) (*N* = 4940). Slope, correlation coefficient, coefficient of repeatability (CR) and *p*‐values are shown in the inset, and the 95% prediction intervals are shown by the dashed lines. Bottom row: Bland–Altman analysis for the HFA (left) and Virtual Field (right) when comparing Tests 2 and 3. The red solid line indicates the mean bias, the red dashed lines indicate the 95% limits of agreement, the blue solid line indicates the regression line (*p*‐value of the slope is shown in the inset), and the black solid line indicates *y* = 0. For the bottom panels, a more positive value indicates that the HFA returned a higher result, and a negative value indicates that the Virtual Field returned a higher result.

**FIGURE 4:**
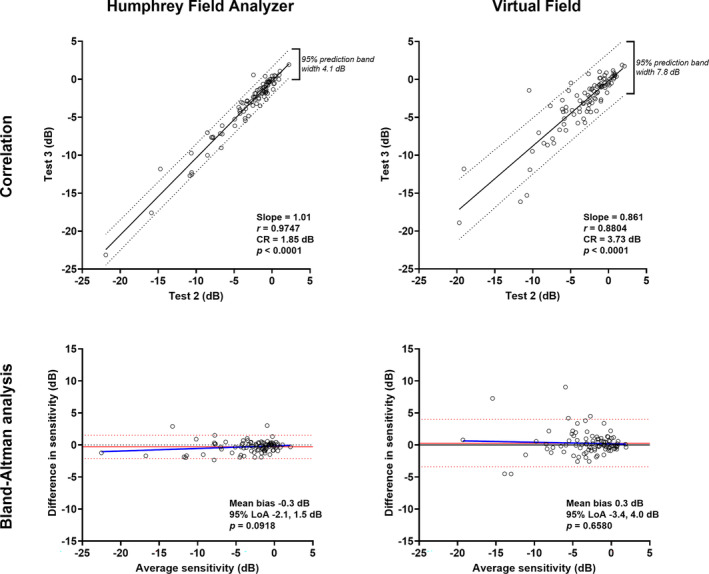
As in Figure 3, but for comparisons of mean deviation between Tests 2 and 3 (*N* = 95).

The correlation was higher for test–retest variability when using the HFA compared to the VF (Pearson correlation *r =* 0.87 vs. *r* = 0.75; intraclass correlation 0.88 vs. 0.72, Figure 3). The Bland–Altman comparisons also showed narrower 95% limits of agreement for the HFA (−6.4 to 5.9 dB) compared to the VF (−7.6 to 8.2 dB). The CR calculated for Tests 2 and 3 was 6.49 dB for the HFA and 7.83 dB for the VF. Similar findings were evident when examining test–retest variability of the mean deviation. The intraclass correlation for the HFA remained high at 0.97, with a slightly lower value for the VF at 0.88. The 95% limits of agreement were similarly narrower when using the HFA (−6.4 to 5.9 dB) compared to the VF (−7.6 to 8.2 dB) (Figure 4). The coefficient of repeatability (CR) for mean deviation was 1.85 dB for the HFA and 3.73 dB for the VF.

### Pointwise visual field defects on probability maps

**FIGURE 5:**
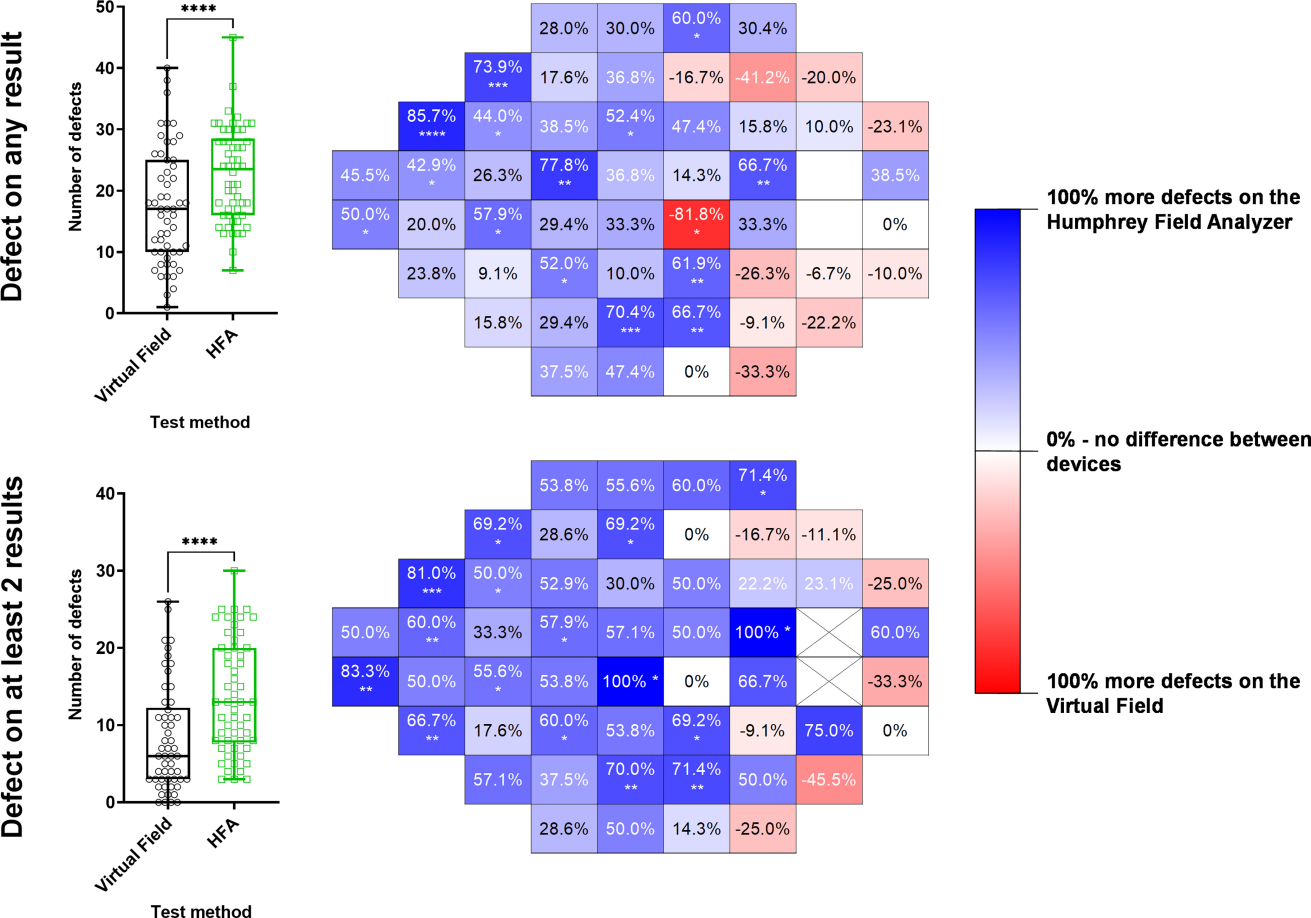
Left: box and whisker (median, interquartile range and full range) distributions for the number of defects detected using the Virtual Field (black) and Humphrey Field Analyzer (HFA) (green) (*N* = 95). Defects were defined as per the instrument's own normative database and were extracted from the pattern deviation map. Each datum point indicates the result from one subject with glaucoma. Right: heat map for relative number of defects detected using each test method across each 24‐2 test location (right eye orientation). A colder/bluer colour indicates a greater proportion of defects detected using the HFA and a warmer/redder colour indicates more defects found using the Virtual Field. The top row indicates results where any of the tests for each subject identified a defect, while the bottom row indicates the results where at least two of the three tests showed a defect.

At the individual level and when comparing the subject to the underlying normative database, the HFA detected, on average, 7 (out of 52) more defects (when at least one result showed a defect) and 5 (out of 52) more defects (when a repeatable defect was required) compared to the VF (*p* < 0.0001) (Figure 5). The results of the McNemar tests at each location showed nasal step, inferior arcuate and superior arcuate regions where the HFA detected more defects compared to the VF. As described in the Methods, the results when empirically derived normative data from the present study were used are shown in Figure S4, which suggested that the VF may detect slightly more defects compared to the HFA. However, given that the current study compared an available iteration of the VF device with the HFA, the theoretical analysis with the empirical normative data is presented in Appendix S1 and is discussed further in the Discussion.

Within‐subject comparisons of the number of defects detected using each device's normative database showed weak correlations for both criteria (*r* = 0.38 when at least one result showed a defect; *r* = 0.31 when a repeatable defect was required; Figure S6).

### Intra‐visit effects of learning and fatigue

Since the subjects conducted all tests at the same visit, it is possible that there may be effects of learning and fatigue on the resultant perimetric outputs, despite allowing breaks between tests. To analyse this, we performed a one‐way ANOVA (with matching by subject) on the mean deviation, pattern standard deviation and pointwise sensitivity outputs for each test and for each subject. This was done in an intra‐test manner (i.e., comparing Tests 1, 2 and 3 when using the same device), rather than strict test order, due to the much higher number of different permutations. The results are summarised in Table 1.

**TABLE 1:** One‐way ANOVA examining the differences in mean deviation, pattern standard deviation and pointwise sensitivities between Tests 1, 2 and 3 for each device (mean difference of 95% confidence interval [CI] and *p*‐value), and the 95% limits of agreement (LoA) from pairwise Bland–Alman analysis.

	Test 1–Test 2			Test 1–Test 3			Test 2–Test 3		
	Mean difference and 95% CI	*p*‐Value	95% LoA	Mean difference and 95% CI	*p*‐Value	95% LoA	Mean difference and 95% CI	*p*‐Value	95% LoA
Humphrey Field Analyzer									
Mean deviation	0.96 (0.69 to 1.24)	<0.0001	−1.23 to 3.16	1.23 (0.92 to 1.55)	<0.0001	−1.28 to 3.75	0.27 (0.05 to 0.49)	0.01	−1.51 to 2.05
Pattern standard deviation	−0.31 (−0.55 to −0.08)	0.006	−2.21 to 1.58	−0.29 (−0.56 to −0.03)	0.03	−2.43 to 1.84	0.02 (−0.20 to 0.24)	0.98	−1.73 to 1.77
Pointwise sensitivities	0.98 (0.85 to 1.12)	<0.0001	−6.28 to 9.25	1.34 (1.20 to 1.48)	<0.0001	−6.12 to 8.79	0.35 (0.23 to 0.48)	<0.0001	−6.10 to 6.81
Virtual Field									
Mean deviation	−0.12 (−0.61 to 0.37)	0.84	−4.04 to 3.81	−0.38 (−0.95 to 0.19)	0.25	−4.93 to 4.17	−0.26 (−0.73 to 0.20)	0.37	−3.98 to 3.45
Pattern standard deviation	−0.06 (−0.28 to 0.16)	0.79	−1.81 to 1.69	−0.03 (−0.29 to 0.23)	0.96	−2.09 to 2.03	0.03 (−0.18 to 0.25)	0.94	−1.69 to 1.76
Pointwise sensitivities	−0.12 (0.27 to 0.03)	0.14	−7.99 to 7.75	−0.43 (−0.59 to −0.27)	<0.0001	−8.92 to 8.06	−0.31 (−0.46 to −0.17)	<0.0001	−8.12 to 7.49

The findings showed significant differences between Tests 1, 2 and 3 (except for the comparison of pattern standard deviation when examining Tests 2 and 3) when using the HFA. The direction of differences suggested a worsening of visual field performance (more negative mean deviation, more positive pattern standard deviation and less positive pointwise sensitivity) with later tests, which could potentially be attributable to the effects of fatigue. The 95% limits of agreement tended to be least wide when comparing Tests 2 and 3, suggesting that there may also be a learning effect, narrowing the difference between test results in comparison with when Test 1 was used as a reference. For the VF results, there were no significant differences between Tests 1, 2 and 3 for mean deviation and pattern standard deviation, though Test 3 showed slightly lower sensitivity values compared to Tests 1 and 2. The differences in 95% limits of agreement between tests were similar for the VF device.

## DISCUSSION

In the present study, we compared an iteration of a new VR perimetry device, the VF, against the HFA. We found that global indices and sensitivity results were moderate to highly correlated between the devices; however, there appeared to be greater intra‐visit test–retest variability and relatively wider limits of agreement when using the VF. In particular, test–retest variability is an important metric for obtaining consistent data relevant for disease diagnosis and its monitoring. There was also an unexplained mean difference between output sensitivities that was not fully explained by the differences in background luminance. There were some statistically, but unlikely clinically, significant differences in reliability metrics. There were differences in the number of visual field defects detected by each instrument, depending on the reference normative data. In combination, these results suggest that outputs between the two devices are not directly interchangeable.

### Comparison of global indices

Mean deviation is a commonly used metric for trend‐based analysis and for staging glaucoma.^33^, ^35^ We found no significant difference between mean deviation results despite some differences in pointwise sensitivities, and both devices were strongly correlated. Therefore, at a given HFA mean deviation level, the VF returns a very similar result. However, as this was a cross‐sectional study, we do not make any inferences regarding longitudinal trends in mean deviation and the stability of its measurement for either device.

There was a systematic difference between devices in terms of the pattern standard deviation, with the VF returning relatively lower values compared to the HFA with increasing levels of pattern standard deviation. This could be explained by the dynamic ranges of the two devices. The VF, with a smaller dynamic range, is less likely to have variation in the pattern deviation result, leading to a lower pattern standard deviation, in comparison with the HFA. Given the difference in pattern standard deviation results between devices, criteria for identifying a significantly elevated pattern standard deviation, such as that suggested by the Ocular Hypertension Treatment Study,^36^ should not be applied to data obtained using the VF. Although this reflects different scales and may not necessarily indicate the severity of the defect, the clinician‐facing nature of the pattern standard deviation metric raises concerns about whether it would be *perceived* as an underestimation of the defect.

### Comparison of test duration and reliability metrics

Test duration was shorter for the VF compared to the HFA. This was likely attributable to the differences in test algorithms. One potential benefit of a shorter test duration is a reduction in the effect of fatigue. Table 1 shows a potential manifestation of this finding. Additionally, Table 1 does not demonstrate a marked learning effect, possibly because the subjects had practice prior to the runs being recorded. However, it is known that within the SITA family, faster algorithms tend to return more variable results (such as comparisons between SITA‐Standard and SITA‐Faster), with recent studies describing trends in variability and exploring reasons contributing to such trends.^20^, ^37^ This may lead to the assumption that the greater test–retest variability in the VF may be attributable to the faster test algorithm. However, since the VF test algorithm remains proprietary information, we cannot directly test this hypothesis. Despite potential differences in measurement variability, Saunders et al.^38^ demonstrated that small differences are unlikely to be significant contributors in the application for detecting disease progression when comparing SITA‐Standard and SITA‐Fast. Real‐world comparison of algorithms from different manufacturers requires further testing to determine the effect of algorithmic differences in disease and progression detection.

The use of automated reliability indices for determining result reliability has been the topic of debate in the recent literature.^22^, ^23^, ^24^, ^25^ Nonetheless, we compared these indices in the present study as they are likely to remain widely reported by commercial, clinical perimetry devices. Both the HFA and VF use the Heijl–Krakau method for determining fixation loss rate.^27^ Fixation losses tended to be higher on the HFA, and the reason for this may be because of the platform that is used. The VR headset can compensate for head movements by shifting the image on the screen. Although the HFA has a head tracker capable of detecting and compensating for some head movements, it may be more susceptible to changes in head position, which could lead to a greater rate of fixation losses. False‐positive rates were also higher on the HFA, albeit the difference was very small, and were unlikely to be clinically significant.

### Comparison of pointwise sensitivity

Output sensitivities in decibels are affected by the maximum and background luminance parameters of the device, and thus, there are differences expected if examining only the output results. Furthermore, in combination with given different retinal adaptation levels at which sensitivity was assessed (due to the different background luminance values), we expected lower output sensitivity values with the VF. The difference was maintained (albeit in the opposite direction) when the equivalent Humphrey Field Analyzer (eqHFA) sensitivity (accounting for luminance range differences) was calculated (Appendix S1). The difference in variability was also similarly maintained. This is in addition to the differences in the visual pathway that were assessed by each perimeter (photopic assessed by the HFA and mesopic by the VF). This reinforces the conclusion that direct, one‐to‐one decibel comparisons between the devices should not be performed, in line with previous recommendations when comparing other perimetric devices.^39^

As mentioned in the Introduction and Methods, there were differences in retinal illuminance (and therefore retinal adaptive state) when tested using each perimeter due to the background luminance. It is possible that further refinements to the VF device could render the background closer to the photopic range without significantly sacrificing a useful dynamic range, but this would require a different experimental design. Even so, because of differences in the physical parameters between devices, a head‐to‐head comparison of HFA and VF using comparable background luminance and dynamic range parameters would be technically challenging.

Test–retest variability is an important parameter for understanding the significance of sensitivity change between tests, the dynamic range of the device and estimating the measurement floor, a concept that is also affected by test parameters.^31^, ^32^ The VF had a higher test–retest variability compared to the HFA in terms of both pointwise sensitivities and mean deviation. For pointwise sensitivity, both devices exhibited heteroscedasticity, with a notable increase in variability below a certain sensitivity level (representing an estimate of the measurement floor); for the HFA, this was approximately 19 dB (consistent with previous studies^31^, ^40^), while for this iteration of the VF device, this was approximately 12–13 dB using their respective scales. Again, the difference in the floor may be attributable to the device's physical range and the background luminance (and therefore the state of retinal adaptation). Additionally, the mesopic test background for the VF may have led to greater variability (representing an intrinsic physical limitation of the device), due to the threshold‐versus‐intensity curve being closer to the de Vries‐Rose region (assuming cone‐mediated detection as described by the work of Simunovic et al.^28^), and thus being limited by quantal fluctuations.^41^, ^42^

### Comparison of statistically significant defects

Given that the iteration of the VF examined in the present study has a clinical printout, the core evaluation was based on its current output, rather than potential differences that could be mitigated with theoretical modifications to the normative database. In other words, using the current clinical output, the VF detects fewer defects compared to the HFA. Our results suggest that improvements in future iterations of the VF device or software could narrow the differences (see Appendix S1).

Apart from differences in normative databases, we initially predicted that the VF would detect more defects due to the use of a mesopic background. Drum et al.^43^ showed that in glaucoma, scotopic sensitivity tends to be more severely affected than photopic sensitivity. Later, Jonas et al.^44^ identified dark adaptation perturbations in glaucoma. Further psychophysical evidence was provided by Fortune et al.^45^ in an electrophysiological study of an animal model of glaucoma. Although the results shown in Appendix S1 appear to support this hypothesis, the small size of the normative database limits our ability to make more conclusive recommendations. Additionally, there was variability in the number of defects identified across devices, which further suggests that the results are not directly interchangeable. In the absence of a greater number of defects identified using this iteration of the VF device, further study in this area and the significance of these differences is therefore essential to test the theoretical framework of scotopic and mesopic sensitivity.

## CONCLUSIONS

The evaluated iteration of the VF VR headset perimeter shows strong correlations with the HFA in terms of mean deviation and sensitivity outputs, but there was a systematic bias in the pattern standard deviation outputs likely reflective of the difference in dynamic range, retinal adaptation and other hardware‐ and software‐related factors. There are significant differences in defect detection and variability characteristics that may be due to different underlying normative databases, physical range and background luminance (thus testing under different levels of retinal adaptation), requiring further investigation and refinement. While there may be practical advantages to using a virtual reality headset perimeter, the effect of differences in intra‐visit test–retest variability and the variability in number of defects between instruments remains unknown, but requires further study in terms of its impact on disease diagnosis and progression monitoring in larger longitudinal studies.

## AUTHOR CONTRIBUTIONS

**Jack Phu:** Conceptualization (equal); data curation (equal); formal analysis (lead); funding acquisition (supporting); investigation (lead); methodology (lead); project administration (equal); supervision (equal); validation (equal); visualization (equal); writing – original draft (lead); writing – review and editing (equal). **Henrietta Wang:** Data curation (equal); investigation (equal); methodology (equal); writing – review and editing (equal). **Michael Kalloniatis:** Conceptualization (equal); formal analysis (equal); funding acquisition (equal); project administration (equal); resources (equal); writing – review and editing (equal).

## FUNDING INFORMATION

The work was supported, in part, by an NHMRC Ideas Grant to MK and JP (1186915). Guide Dogs NSW/ACT provided funding for the clinical services enabling data collection for this study and provides salary support for HW. Virtual Fields provided a headset unit to undertake visual field testing. The funding organisations had no role in the design or conduct of this research.

## CONFLICT OF INTEREST STATEMENT

Virtual Fields provided a headset unit to undertake visual field testing. The organisation had no role in the design or conduct of this research, or the decision to publish.

## Supporting information


Appendix S1.
