# Supplementary material for: Comparing a head-mounted virtual reality perimeter and the Humphrey Field Analyzer for visual field testing in healthy and glaucoma patients
Source: Ophthalmic Physiol Opt. 2023 Oct 6;44(1):83–95. doi: 10.1111/opo.13229 (PMC10952716; doi:10.1111/opo.13229)
Supplement: Supplementary file 1 — Comparing a head mounted viral reality perimeter and the Humphrey Field Analyzer for visual field testing in glaucoma patients [file 44402_2024_4401010_MOESM1_ESM.docx]

**SUPPLEMENTARY MATERIAL: Comparing a head mounted viral reality perimeter and the Humphrey Field Analyzer for visual field testing in glaucoma patients**

**Accounting for differences in instrument dynamic range**

Since decibel (dB) is a measure of attenuation in perimetry, its value and scale are related to the maximum output luminance and background luminance of the device. With differences in maximum and background luminance values between devices, the decibel outputs are not directly interchangeable. The relationship between threshold luminance and output decibel is shown by the following equation.^22^

$$Threshold luminance \Delta L \left( cd.m^{-2} \right)= \frac{{\Delta L}_{max}}{{10}^{\left( \frac{dB}{10} \right)}}$$

Where dB is the output decibel value and Δ*L_max_* is the maximum luminance. Thus, replacing these values with the background luminance values from the Humphrey Field Analyzer (3183 cd.m^-2^) and the Virtual Field (87 cd.m^-2^) returns the following equations.

$$Threshold luminance \Delta L \left( cd.m^{-2} \right)= \frac{3183}{{10}^{\left( \frac{HFA-dB}{10} \right)}}$$

$$Threshold luminance \Delta L \left( cd.m^{-2} \right)= \frac{87}{{10}^{\left( \frac{VF-dB}{10} \right)}}$$

Where HFA-dB and VF-dB are the decibel outputs for the Humphrey Field Analyzer and Virtual Field devices, respectively. By using these equations, we can obtain an ‘equivalent’ Humphrey Field Analyzer dB value for a given Virtual Field dB value, as the threshold luminance has been equated. The goal of this analysis was to facilitate a comparison between output dB values from both devices for interpretation, in methods similar to that described by Parodi et al.^23^ The ‘equivalent’ Humphrey Field Analyzer (eqHFA) dB for a given Virtual Field dB value was returned by the following equation.

$$eqHFA=10 \times{log}_{10}\left( \frac{3183\times{10}^{\frac{VF-dB}{10}}}{87} \right)$$

*Results*

The results of these analyses are shown in Supplementary Figure 1, which are analogous to the analyses shown in Figure 2 in the main manuscript. The eqHFA decibel results were, expectedly, higher, than the base Virtual Field decibel level. The mean difference as determined by Bland-Altman analyses were 9.5 dB and 9.2 dB higher on the Virtual Field (eqHFA) across conditions of all test results and after excluding points reaching the floor, respectively. This was notably lower than the predicted difference, given that the difference in background luminance between devices was almost two log units (10 cd.m^-2^ versus 0.218 cd.m^-2^). The intraclass correlations were similar to the results reported in the manuscript and remained low (0.3459 when including all points and 0.4279 when excluding the floor).


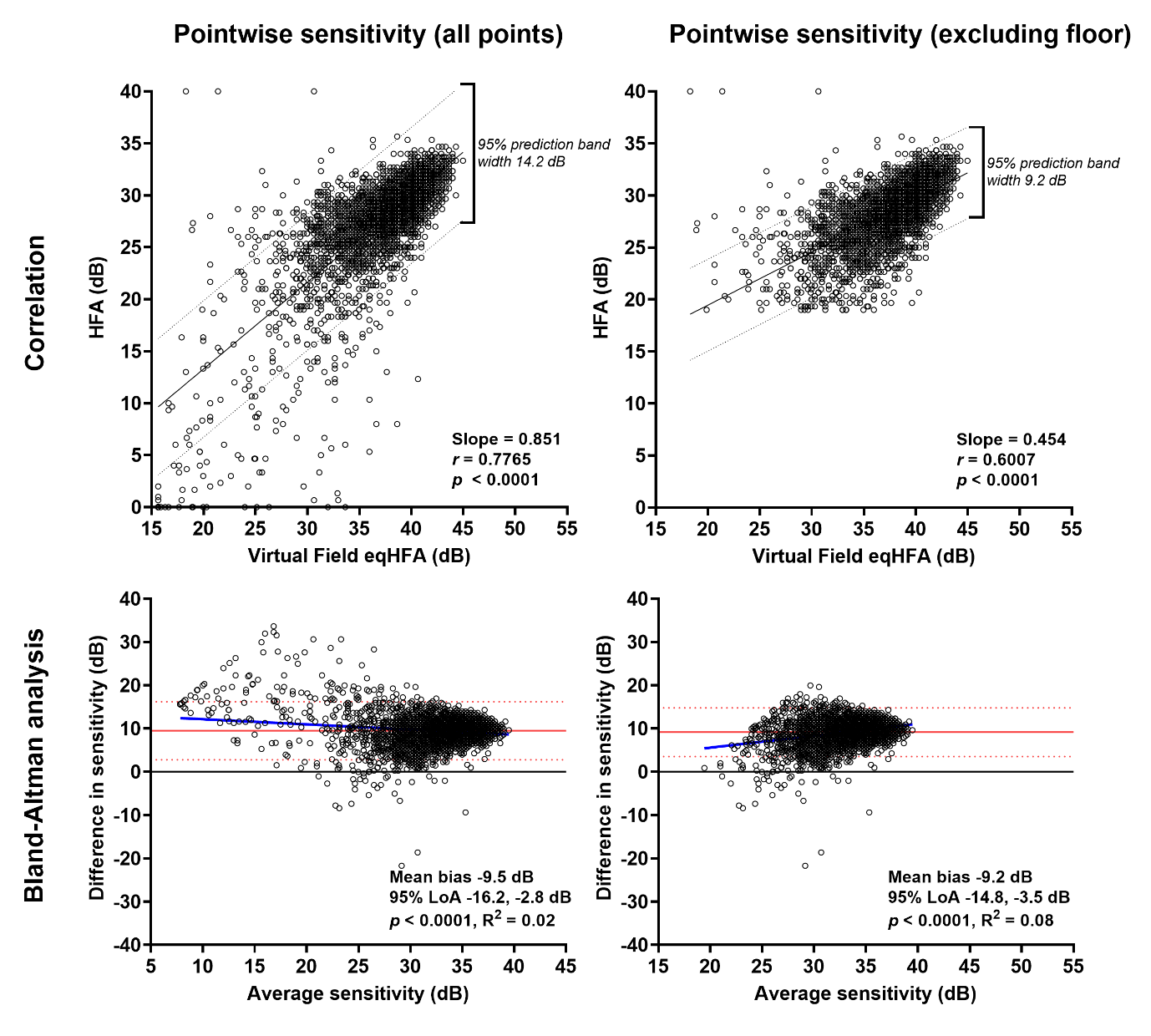


**Supplementary Figure 1:** Top row: correlations between Humphrey Field Analyzer (HFA) and Virtual Field equivalent Humphrey Field Analyzer (eqHFA) pointwise sensitivity inclusive of all points (left) (N = 4,940) and when points reaching the HFA floor (< 19 dB) were excluded (right) (N = 4,617). Slope, correlation coefficient and *p*-values are shown in the inset, and the 95% prediction intervals are shown by the dashed lines. Bottom row: Bland-Altman analyses for pointwise sensitivity inclusive of all points (left) and when points reaching the HFA floor (< 19 dB) were excluded (right). The red solid line indicates the mean bias, the red dashed lines indicate the 95% limits of agreement, the blue solid line indicates the regression line (*p*-value of the slope is shown in the inset), and the black solid line indicates y =0. For the bottom panels, the differences were eqHFA – HFA, such that a positive y-axis value indicates a higher equivalent Humphrey Field Analyzer (eqHFA) sensitivity value found on the Virtual Field, and vice versa.

*Discussion: correcting Virtual Field outputs into ‘equivalent’ Humphrey Field Analyzer sensitivities*

The purpose of the eqHFA was to account for the difference in background luminance and ‘convert’ the Virtual Field decibel output into what might be expected if using the luminance range of the Humphrey Field Analyzer.^23^

One reason for the difference in sensitivity not meeting the predicted level is because of the change in effective retinal illuminance, or the adaptive state of the eye. As the Virtual Field background luminance is at the mesopic level of retinal adaptation, the threshold-versus-intensity relationship may not be on the Weber slope.^30-32^ If considering the models proposed by Kalloniatis and Harwerth,^31^ the mesopic background, especially in the context of assessing a disease state such as glaucoma, would return a < 1:1 relationship between sensitivity and background. Therefore, a one-to-one conversion in actual and predicted decibel values is not appropriate, and a lesser change is expected.

**Normative comparisons with an empirically derived normative database**

The differences between the devices’ underlying normative databases means that the significance of defects at different probability levels are not necessarily equivalent. In this additional analysis, we developed normative ranges using the healthy subjects examined in the present study.

We have previously reported on suggestions for the number of subjects recommended for a normative database.^24^ In the present study, we had a slightly smaller number of healthy subjects, but each subject underwent repeat tests, and the subjects were within an age-similar group (so age correction was not performed). At each test location, the 5^th^ percentile (lowest) sensitivity result was used to denote significant defect equivalent to the *p* < 0.05 result found on commercial perimeters. This analysis was limited to the *p* < 0.05 level, as the sample size was likely inadequate for determining lower limits, like *p* < 0.01. The derived lower limits are shown in Supplementary Figures 2 and 3.


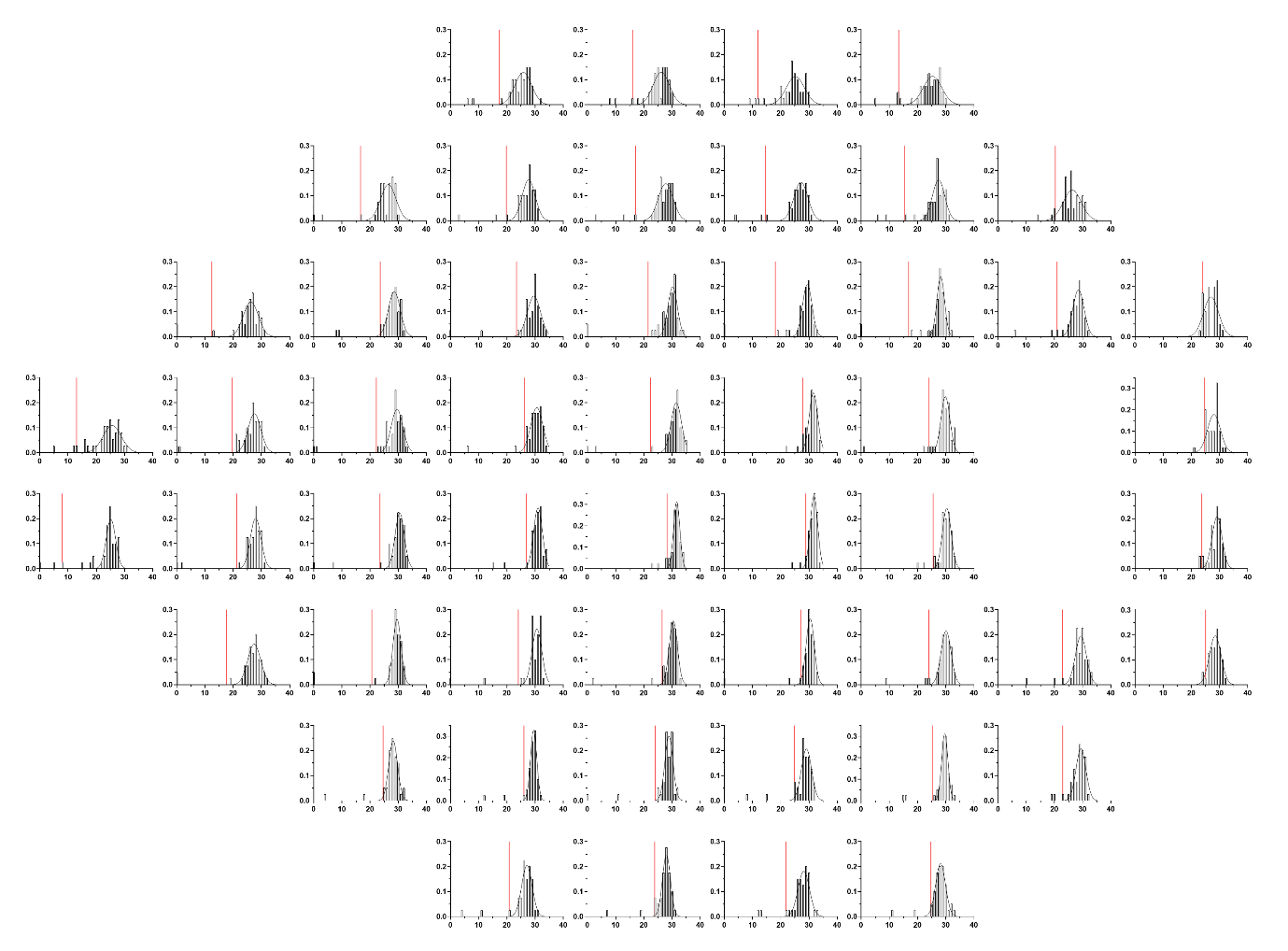


**Supplementary Figure 2:** Sensitivity distributions fitted with Gaussian functions for the healthy cohort within the present study at each 24-2 test location (except for the physiological blind spot) for the Humphrey Field Analyzer (N = 95). The sensitivity steps are in 1 dB increments. The red line indicates the lower 5^th^ percentile of the distribution.


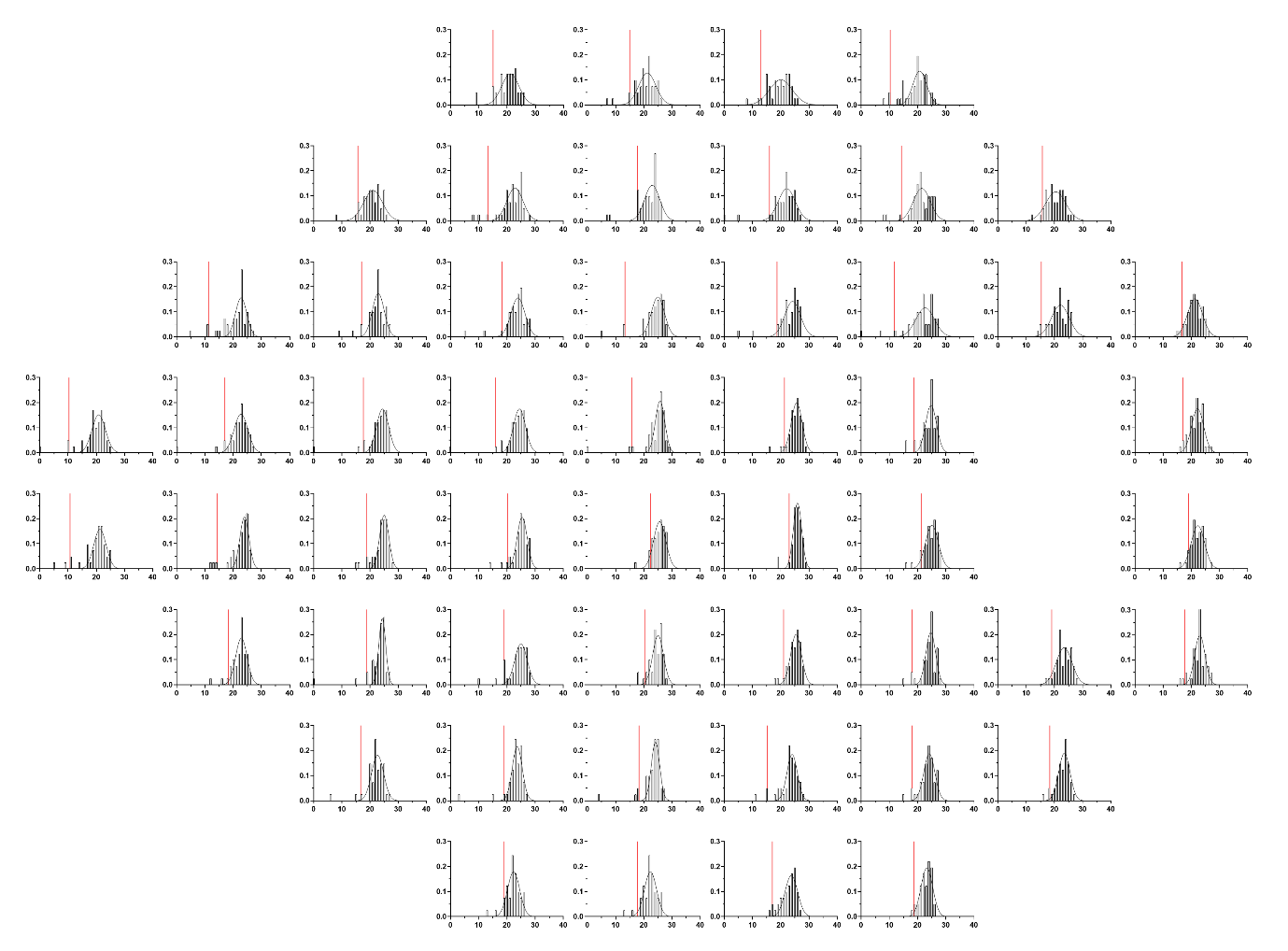


**Supplementary Figure 3:** Sensitivity distributions fitted with Gaussian functions for the healthy cohort within the present study at each 24-2 test location (except for the physiological blind spot) for the Virtual Field (N = 95). The sensitivity steps are in 1 dB increments. The red line indicates the lower 5^th^ percentile of the distribution.

*Results of empirical normative analysis*

The results of pointwise defects relative to the normative data generated by the normal subjects (n=41) in the present study are shown in Supplementary Figure 4. Notably, the results were the opposite to that shown in Figure 5 of the main manuscript, with most locations, especially in the nasal step, superior arcuate and inferior arcuate regions, showing more defects on the Virtual Field compared to the Humphrey Field Analyzer. The mean difference in number of defects was 4 (out of 52) points and 2 (out of 52) points favouring the Virtual Field for any defect and for a repeatable defect, respectively.


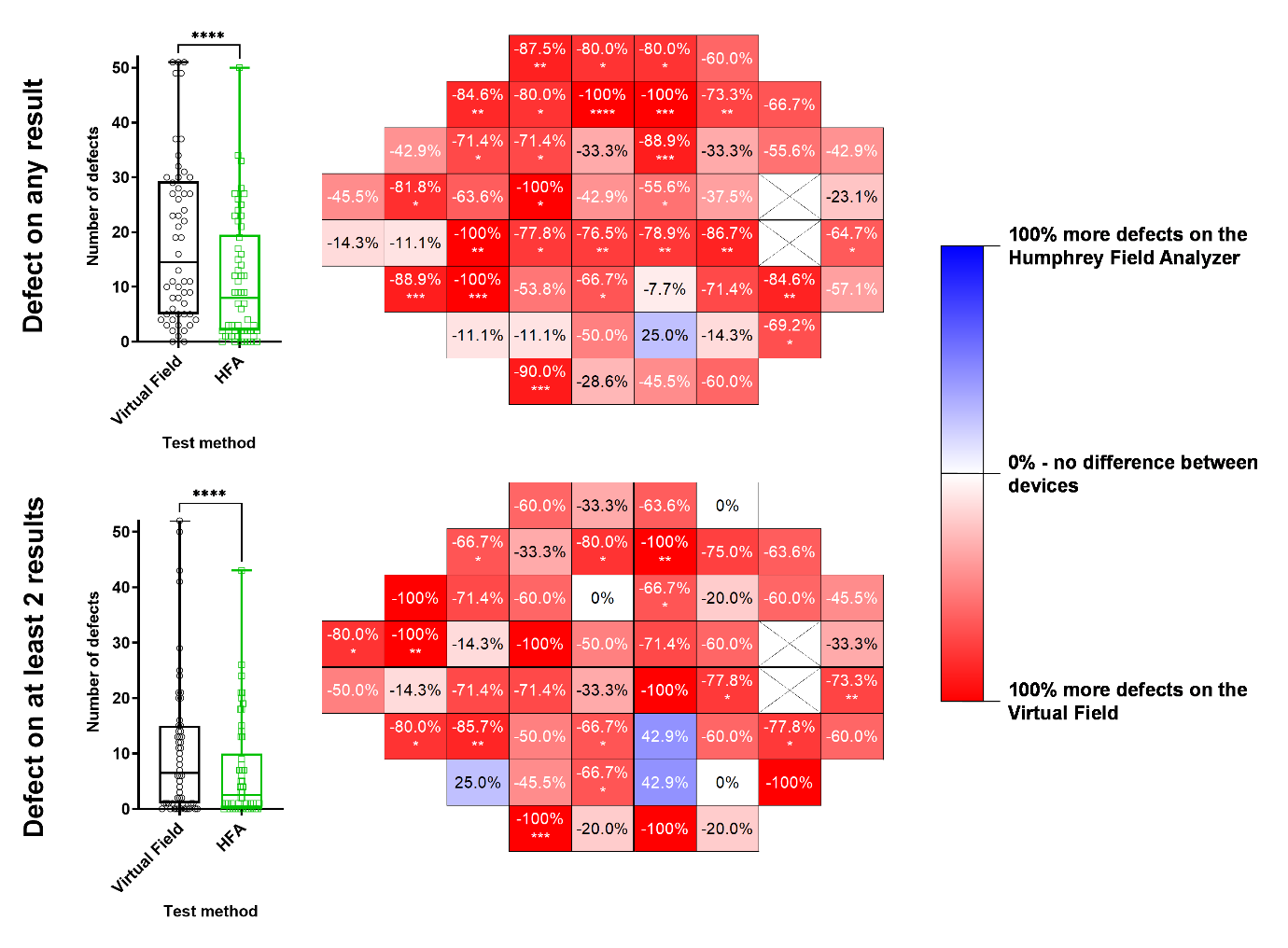


**Supplementary Figure 4:** Left: box and whisker (median, interquartile range and full range) distributions for number of defects detected using the Virtual Field (black) and Humphrey Field Analyzer (HFA) (green), when determined using the data from the healthy subjects of the present cohort (N = 95). Defects were defined as per the instrument’s own normative database and were extracted from the pattern deviation map. Each datum point indicates the result from one subject with glaucoma. Right: heat map for relative number of defects detected using each test method across each 24-2 test location (right eye orientation). A colder/bluer colour indicates a greater proportion of defects detected using the HFA, and a warmer/redder colour indicates more defects found using the Virtual Field. The top row indicates results where any of the tests for each subject identified a defect, and the bottom row indicates the results where at least two of the three tests showed a defect.

**Test duration and reliability indices**


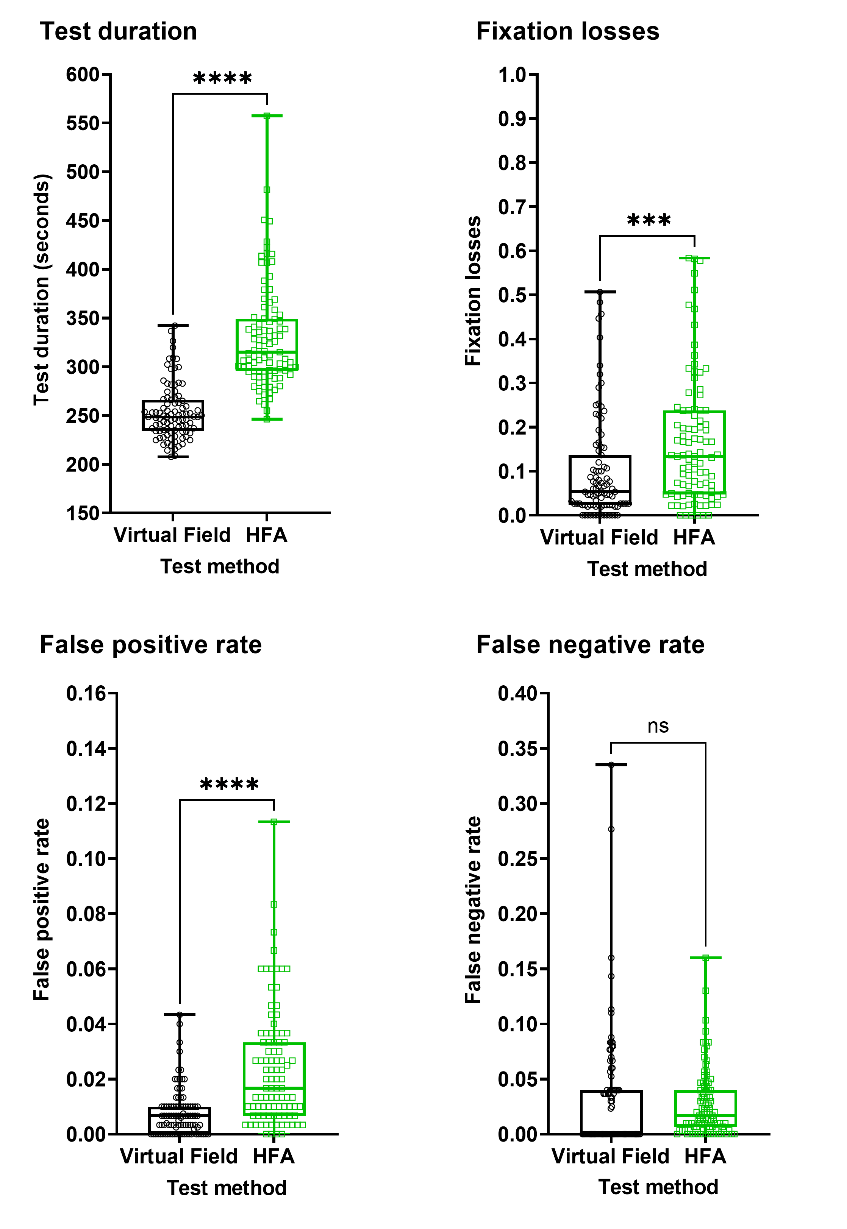


**Supplementary Figure 5:** Box and whisker (median, interquartile range and full range) distributions of test duration, fixation loss, false positive rate, and false negative rate for Virtual Field (black) and Humphrey Field Analyzer (HFA) (green) (N = 95). Bars indicate the level of significance of statistical comparisons between test method (ns = not significant; ***, *p* < 0.001; ****, *p* < 0.0001).

**Within-subject comparisons of number of defects detected between both devices’ normative database**


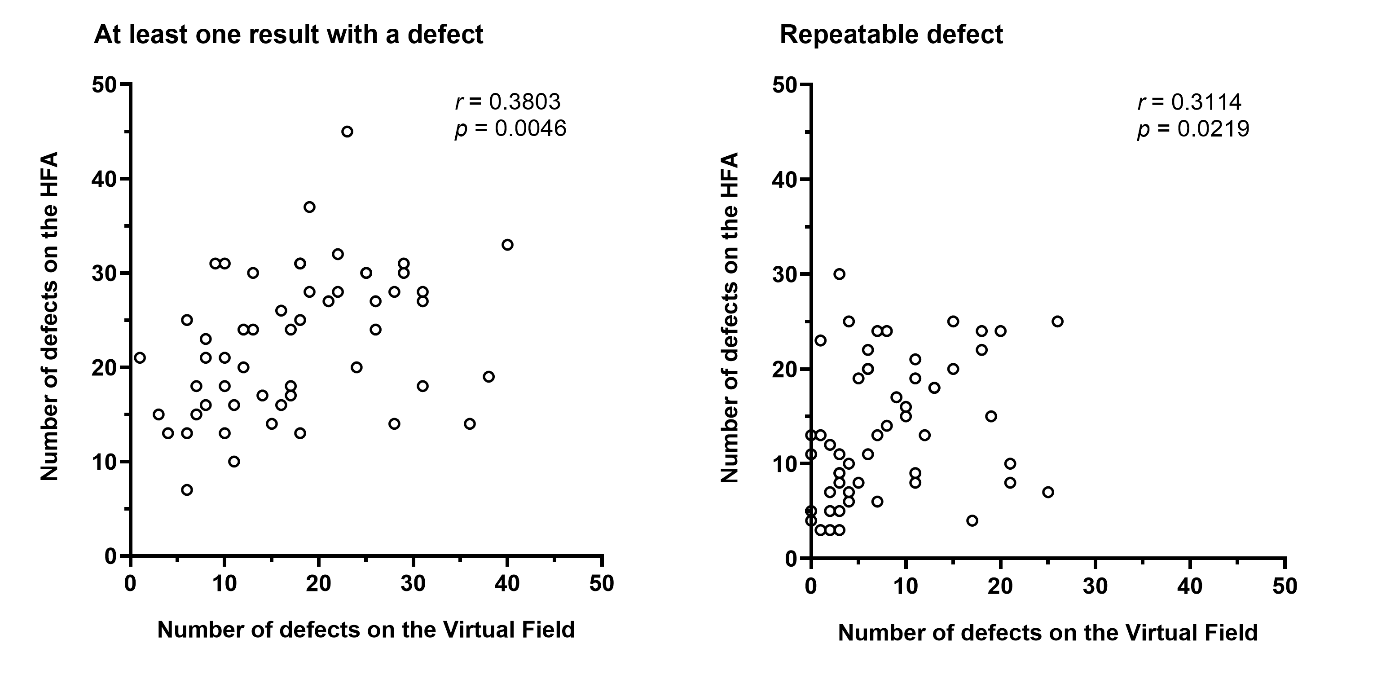


**Supplementary Figure 6:** Correlations between the number of pointwise defects found using the Humphrey Field Analyzer (HFA) and the Virtual Field for the criteria requiring a defect found on any result (left) or a repeatable defect (right) for the glaucoma subjects (N = 54). Each datum point represents one subject with glaucoma, with the insets showing the correlation and *p*-value.
